# The many disguises of the signalling endosome

**DOI:** 10.1002/1873-3468.13235

**Published:** 2018-09-17

**Authors:** David Villarroel‐Campos, Giampietro Schiavo, Oscar Marcelo Lazo

**Affiliations:** ^1^ Department of Neuromuscular Diseases UCL Queen Square Institute of Neurology University College London UK; ^2^ UK Dementia Research Institute at UCL London UK; ^3^ Discoveries Centre for Regenerative and Precision Medicine University College London Campus UK

**Keywords:** axonal transport, endosomes, neurotrophins, signalling

## Abstract

Neurons are highly complex and polarised cells that must overcome a series of logistic challenges to maintain homeostasis across their morphological domains. A very clear example is the propagation of neurotrophic signalling from distal axons, where target‐released neurotrophins bind to their receptors and initiate signalling, towards the cell body, where nuclear and cytosolic responses are integrated. The mechanisms of propagation of neurotrophic signalling have been extensively studied and, eventually, the model of a ‘signalling endosome’, transporting activated receptors and associated complexes, has emerged. Nevertheless, the exact nature of this organelle remains elusive. In this Review, we examine the evidence for the retrograde transport of neurotrophins and their receptors in endosomes, outline some of their diverse physiological and pathological roles, and discuss the main interactors, morphological features and trafficking destinations of a highly flexible endosomal signalling organelle with multiple molecular signatures.

## **Abbreviations**

**ALS**, Amyotrophic lateral sclerosis

**APC**, adenomatous polyposis coli

**Aβ**, amyloid beta

**BDNF**, brain‐derived neurotrophic factor

**CMT**, Charcot‐Marie‐Tooth disease

**CREB**, cAMP response element‐binding protein

**CTB**, cholera toxin B

**DIC**, dynein intermediate chains

**DLC**, dynein light chains

**DRGs** , Dorsal root ganglia

**GAPs**, GTPase‐activating proteins

**GCPs**, granule cell precursors

**GDNF**, glial‐derived neurotrophic factor

**GEFs**, guanine nucleotide exchange factors

**GSK3ß**, glycogen synthase kinase 3 beta

**HOPS**, homotypic fusion and sorting complex

**Htt**, huntingtin

**MAPKs**, mitogen‐activated protein kinases

**MVBs**, multivesicular bodies

**NGF**, nerve growth factor

**NT3**, neurotrophin‐3

**NT4/5**, neurotrophin‐4/5

**p75** ^**NTR**^, p75 neurotrophin receptor

**PI3K**, phosphoinositide 3‐kinase

**PLCγ**, phospholipase C‐gamma

**polyQ**, polyglutamine

**PtdIns(3)P**, phosphatidylinositol‐3 phosphate

**pTrkB**, phosphorylated TrkB

**SOD1**, superoxide dismutase 1

**Trk**, tropomyosin‐related kinase receptors

## Where trafficking meets signalling

### How to propagate a retrograde signal

The complexity and high polarisation of neurons impose a mighty challenge for the precise and timely delivery of material to different subcellular compartments, which is needed for differentiation and homeostatic control. Proper function of the nervous system relies on the ability of neurons to integrate events occurring across their different domains to regulate their metabolism, excitability, and morphology 1, 2, 3. A very clear example of this challenge is how neurotrophic factors secreted by target cells are sensed by axon terminals, and how their signals are propagated towards the cell body to have an impact on gene expression, synapse formation, dendritic branching, survival, or targeted cell death 4.

Neurotrophins constitute a family of homodimeric ligands that includes nerve growth factor (NGF), brain‐derived neurotrophic factor (BDNF), neurotrophin‐3 (NT3), and neurotrophin‐4/5 (NT4/5). They bind two different types of receptors, tropomyosin‐related kinase receptors (Trk) and the p75 neurotrophin receptor (p75^NTR^). Trk receptors bind specific neurotrophins; TrkA binds preferentially NGF, TrkB binds BDNF and NT‐4/5, and TrkC binds NT‐3. When activated, they promote cell survival, axonal growth, and dendritic branching through their interaction with specific neurotrophins and downstream activation of signalling cascades, comprising phosphoinositide 3‐kinase (PI3K)‐Akt pathway, mitogen‐activated protein kinases (MAPKs) and phospholipase C‐gamma (PLCγ) 5. In contrast, p75^NTR^ binds all neurotrophins with similar affinity, as well as their unprocessed forms called proneurotrophins, and other ligands 6. Due to its lack of intracellular catalytic activity, p75^NTR^ depends on ligand‐induced recruitment of adaptors and coreceptors to exert a wide range of diverse functions, such as potentiating TrkA prosurvival pathways, synergising with sortilin to induce cell death, or interacting with NogoR to induce growth cone collapse 7, 8, 9.

Propagation mechanisms of retrograde neurotrophic signalling have been extensively studied. Rita Levi‐Montalcini observed that radiolabelled NGF accumulates in sympathetic ganglia after subcutaneous or systemic injection, suggesting that the ligand is actively transported from the periphery to neuronal somata 10. In a follow‐up study, Hendry and colleagues demonstrated in 1974 that iodinated NGF injected into the interior eye chamber is taken up by the sympathetic terminals, retrogradely transported to the superior cervical ganglion in a process requiring microtubules, and accumulates in cell bodies for up to 16 h 11.

The discovery that, after ligand‐induced internalisation, TrkA and TrkB receptors are rapidly transported from axon terminals to soma 12, 13, together with the evidence showing that receptors engage specific signalling pathways from postendocytic compartments 14, 15, enabled the formulation of the ‘signalling endosome’ hypothesis, coined by the group of William Mobley 16, 17. According to this model, internalised receptors together with their cargos are transported in specialised endosomes, where they are able to interact with different signalling molecules to regulate local events in axon and dendrites, as well as long distance effects in the cell body, including gene expression 18.

In the last 20 years, extensive research using *in vitro* compartmentalised cultures and *in vivo* models, high‐resolution fluorescence microscopy, electron microscopy and biochemistry, has permitted the detailed analysis of retrograde signalling carriers distribution, composition, and function. Two methodological approaches have particularly furthered our understanding of these organelles. The first is the use of time‐lapse fluorescence microscopy to follow endosomes, analyse their biogenesis, the frequency and speed of retrograde transport, and how the latter is affected under pathological conditions 19, 20, 21, 22, 23. Second, subcellular fractionation, as well as affinity purification methods targeting retrograde signalling carriers, has shed light on their molecular signatures, including the machinery that regulate their transport, specific organelle markers, transported receptors, and associated signalling complexes 24, 25, 26, 27.

### Axonal transport machinery and retrograde signalling

The retrograde transport of neurotrophins and their receptors relies on the polarised distribution of microtubules in axons. Axonal microtubules are uniformly oriented with their plus‐end facing towards the distal axon. Distal dendrites display a similar orientation of microtubule, whereas a mixed distribution can be found in proximal dendrites 28, 29. Molecular motor complexes recognise the lattice of this organised microtubule array and drive directional transport, with most kinesins moving cargoes towards the microtubules plus‐end and cytoplasmic dynein towards the minus‐end 30. Based on the directionality of their movement, the retrograde axonal neurotrophic signalling therefore depends on cytoplasmic dynein transport 31. The dynein complex is formed by six components, each of them present as dimers: the dynein heavy chain (DHC), the intermediate chains (DIC), the light intermediate chains (DLIC), and three light chains (DLC). The dynein complex interacts with the dynactin complex, which is necessary for dynein activity 32. Signalling endosomes carrying activated TrkA associate with the neuron‐specific variant DIC‐1B 33, and upon NGF or BDNF stimulation, DIC is phosphorylated by ERK1/2, a kinase‐activated downstream to Trk receptors, promoting the recruitment of the cytoplasmic dynein complex to signalling endosomes 34. Signalling endosomes also associate with the DLC Tctex1, although it is presently unclear whether this process is activity‐dependent 35. In cortical neurons, TrkB‐containing endosomes use the dynein adaptor Snapin, which interacts with DIC and recruits the dynein complex. In a *Snapin* knockout model, the retrograde transport of TrkB is reduced, negatively affecting BDNF signalling in the soma 36. However, the finding that axonal transport of TrkB is not completely abolished in neurons lacking Snapin indicates that multiple adaptors recruit cytoplasmic dynein on signalling endosomes.

To trigger the neurotrophic retrograde signalling, Trk receptors first need to reach the axonal tip. The delivery of TrkB from the soma to the distal axon depends on kinesin‐1, which binds to a complex constituted by collapsin response mediator protein 2, Slp1 and Rab27B, in cultured hippocampal neurons 37. Alternatively, TrkB can also associate in the same cellular model to c‐Jun NH_2_‐terminal kinase‐interacting protein 3 (JIP3), which directly binds kinesin‐1 light chain, and mediates the anterograde transport of TrkB in axons, but not in dendrites 38. In sensory neurons, the anterograde transport of TrkA is carried out in Rab3‐positive carriers by the kinesin‐3 family member, KIF1A. Dorsal root ganglia (DRGs) from a *Kif1a* ^+/−^ mouse model exhibit progressive TrkA‐positive sensory neuron loss, and develop sensory neuropathy 39.

The transport of signalling endosomes also requires an intact actin cytoskeleton. For instance, in motor neurons in culture, the trafficking of signalling endosomes labelled with a nontoxic fragment of the tetanus toxin (H_C_T), is impaired after treatment with Latrunculin B, an inhibitor of actin polymerisation. In agreement with this, the retrograde transport of these endosomes relies on the coordination between cytoplasmic dynein and myosin Va 40. The aforementioned study takes advantage of an interesting feature of the tetanus toxin (TeNT); namely, that TeNT and its binding fragment H_C_T are transported in axonal neurotrophic signalling endosomes 22, 41. Therefore, fluorescent versions of these bacterial proteins are excellent tools to monitor axonal transport of neurotrophins and their receptors 42. TeNT is not the only virulence factor or pathogen transported in this way, in fact, the signalling endosome pathway is a main portal for neurotrophic viruses and virulent factors targeting the central nervous system 43.

Axonal transport and signalling endosome dynamics are coordinated by Rab GTPases, which are master regulators of intracellular membrane trafficking. They function as molecular switches, cycling between an active, GTP‐bound, and an inactive, GDP‐bound states. Since their intrinsic GTPase activity is very low, the active state is promoted by guanine nucleotide exchange factors (GEFs) and the inactive state is mediated by GTPase‐activating proteins (GAPs) 44. During the Rab cycle, Rab GTPases change their cellular distribution, with the GTP‐bound state thought to associate to the membrane of the target organelle, whilst the GDP‐bound state remains cytosolic, although evidence for a GTP‐independent membrane recruitment is emerging 45. A subset of Rab interactors binds preferentially to the GTP‐bound state, acting as downstream effectors of the Rab activation cascade. Interestingly, some of the Rab effectors are GEFs or GAPs of other Rabs, enabling the interaction between different organelles and the progression of cargoes from one to another 46.

Upon internalisation, neurotrophin receptors are sorted to the endocytic pathway, therefore Rab GTPases regulating the endocytic network are pivotal for neurotrophic signalling. Rab5 is a main determinant of early endosome identity, regulating their motility and homotypic fusion, while Rab7 controls the maturation of late endosomes, their trafficking, and fusion to lysosomes, among other functions 47. In the endocytic pathway, Rab5 recruits Vps34, a lipid kinase that generates phosphatidylinositol‐3 phosphate (PtdIns(3)P), and Rabaptin‐5, which in turn stabilise the Rab5 GEF Rabex‐5, generating a Rab5‐GTP positive feedback loop. Rabex‐5 and PtdIns(3)P are required for the association of Mon1, and the Mon1‐Ccz1 complex to early endosomes. This complex has been shown to act as Rab7 GEF, and together with the Rab5 effector homotypic fusion and sorting complex (HOPS), recruits Rab7 to the endosomal membrane. Additionally, Mon1 interrupts the Rab5 positive feedback loop, enabling the exchange of Rab5 for Rab7 48. This process is known as Rab conversion and contributes to the mechanism by which early endosomes mature into late endosomes 49. Rab conversion also exemplifies how Rab GTPases define the identity of subcellular compartments 50. Which Rab GTPases, GEFs, GAPs, and effectors characterise the retrograde signalling carriers is one of the key aspects defining their identity and will be discussed below.

The subcellular localisation of neurotrophin receptors is crucial for the proper regulation of their downstream signalling. In this regard, neurotrophic signalling controls the trafficking of its own receptors. An example of this can be found during migration of cerebellar granule cell precursors (GCPs), which occurs along a BDNF gradient. In response to this gradient, activated TrkB receptors carried by signalling endosomes accumulate at the leading edge of GCPs, which is oriented towards the BDNF source. TrkB phosphorylation induces the release of BDNF from GCPs, generating an autocrine loop and amplifying the BDNF gradient 51. It has also been shown that in cultured hippocampal neurons stimulated with BDNF, activated TrkB receptors interact with the transmembrane protein retrolinkin, driving endocytosis of phosphorylated TrkB (pTrkB) and sorting to early endosomes positive for the Rab5 effector APPL1 52. Retrolinkin also recruits endophilin A1 to these signalling endosomes, which allows the downstream activation of ERK1/2 and boosts dendritic outgrowth 52. Dendritic branching is also regulated by BDNF in hippocampal neurons, where activated TrkB accumulates in Rab11‐positive recycling endosomes. BDNF‐triggered TrkB activation raises the levels of Rab11‐GTP in dendrites, increasing both the size and the number of stationary recycling endosomes. As a consequence, TrkB levels in dendrites are increased, sensitising neuron to BDNF and promoting dendritic branching 53.

Neurotrophic signalling regulates multiple processes, and neurotrophin receptors exhibit different destinations. The maintenance of cellular homeostasis and survival rely on the ability of neurons to coordinate the journey of these receptors in the endocytic pathway and their sorting after reaching the cell body. In this review, we will briefly present the different cellular roles of retrograde neurotrophic signalling and some key examples of pathological dysfunction in neurodegenerative diseases. We will then discuss the identity of the signalling endosome, its composition, and destinations.

## Retrograde neurotrophic signalling in physiology and pathology

### Physiological roles

The regulation of neuronal survival was the first function originally described for neurotrophic factors, and is intrinsically linked to their discovery 54. During their studies monitoring neuronal death during embryonic development in chick embryos, Viktor Hamburger and Rita Levi‐Montalcini observed that after removal of the wing bud, massive neuronal degeneration takes place in the brachial ganglion innervating the ipsilateral side, at the time when axonal projections reach their targets. They concluded that ‘adequate connections with the periphery are necessary for the maintenance of sensory neurons’ 55. These set of experiments gave rise to the neurotrophic factor theory, which states that during development, neurons are overgenerated, hence in order to survive, they compete for target‐derived trophic factors that are supplied in limited availability 56. Further experiments led to the isolation of NGF as the factor‐promoting survival and maintenance of sympathetic neurons 57, 58. Knockout mice models confirmed those observations, and expanded the neurotrophic factor theory to other members of the neurotrophic family. For instance, NGF and TrkA null mice show an extensive reduction in total neuron number in DRGs and trigeminal ganglion after birth, with almost complete absence of sympathetic ganglia after 10 days 59, 60. BDNF and TrkB knockout models exhibit overt neuronal loss in the vestibular ganglion, as well as moderate but significant neuronal death in DRGs 61, 62. Finally, NT3 null models revealed a marked reduction in proprioceptive DRG neurons, with a concomitant impairment of muscle spindle development 63, a role that is independent of NT3 signalling through TrkC 64. At the molecular level, internalisation and retrograde trafficking of the NGF/TrkA complex are required for the promotion of neuronal survival 65. Distal NGF stimulation of DRGs or sympathetic neurons in compartmented cultures triggers the phosphorylation and activation of the transcription factor cAMP response element‐binding protein (CREB) in the cell body, 66, 67. Phosphorylated CREB induces the expression of the antiapoptotic protein Bcl‐2, and in this way promotes neuronal survival 68. NGF/TrkA retrograde signalling, acting through ERK5, also promotes the expression and activation of the transcription factor MEF2D, which in turn drives the expression of Bcl‐w, another member of the bcl‐2 family with antiapoptotic activity 69, 70. Interestingly, upon target innervation, NGF enhances the expression of TrkA in sympathetic neurons, establishing a positive feedback loop that enhances prosurvival signalling, and at the same time releasing BDNF and NT4, which act as paracrine apoptotic cues that induce death of neighbouring cells through p75^NTR^ 71.

In addition to promoting neuronal survival, the retrograde neurotrophic signalling also regulates other cellular processes, including the control of axonal growth. NGF triggers axon elongation when applied to distal axons of sympathetic neurons, whereas NGF withdrawal induces axon collapse even if it is kept in the somatic compartment 72. In knockout models, the analysis of the effect of neurotrophins on axonal growth requires the ability to discern phenotypic changes independently of their survival roles. This daunting task has been achieved by studying NGF‐ and TrkA‐deficient mice in a *Bax* ^*−/−*^ background, in which sympathetic and DRG neurons overcome cell death in spite of the absence of NGF/TrkA signalling. In these models, some sympathetic innervations are completely absent, while others are partially spared, or not changed at all; this marked heterogeneity depends on the target organs and their different requirements for NGF‐dependent sympathetic innervation 73. Accordingly, the superficial cutaneous innervation of DRG neurons is absent; however, the projections targeting the dorsal horn of the spinal cord are preserved 74. Specific transcription factors acting downstream of NGF regulate axon growth independent of survival effects; this is the case of nuclear factor of activated T cells, serum response factor, and early growth response 3 75, 76, 77.

In addition to retrograde signalling, NGF‐dependent axon elongation also requires local signalling, such as the activation of PI3K at the growth cone, which inactivates glycogen synthase kinase 3 beta (GSK3ß) and regulates cytoskeletal dynamics through the microtubule plus‐end binding protein adenomatous polyposis coli (APC) 78. Interestingly, both NGF and NT3 promote axon growth in sympathetic neurons through TrkA; however, the effect of NT3 is restricted to proximal elongation across the vasculature, whilst distal elongation and target innervations depend on NGF. This spatially controlled regulation is determined by the distinct TrkA signalling triggered by NT3 and NGF, since NT3 is unable to induce TrkA internalisation and signalling from endosomal compartments 79. Conversely, TrkA activation by NGF induces the calcineurin‐dependent dephosphorylation of dynamin 1, which in turn mediates TrkA endocytosis, a crucial step necessary for the induction of axon growth 80.

Dendritic growth and synapse maintenance are also regulated by retrograde neurotrophin signalling. Quantitative morphometric analysis has shown that superior cervical ganglion neurons innervating larger targets elaborate more complex dendritic arborisations 81, and after axonal crush, their dendrites accumulate large varicosities in the distal segment, altering their morphology 82. In addition, *in vivo* NGF stimulation in mice increases sympathetic neurons dendritic length, branching, and number of primary dendrites 83.

Nerve growth factor‐TrkA signalling also modulates synapse formation and maintenance. Indeed, a subpopulation of NGF‐TrkA signalling endosomes originated in distal axons of postganglionic neurons, are retrogradely transported into dendrites, where TrkA‐dependent MAPK activity induces postsynaptic density clustering. Activation of TrkA in axonal signalling endosomes is also essential for the maintenance of synapses between pre‐ and postganglionic neurons, both *in vitro* and *in vivo* 84, 85. Taken together, the control of target innervation, and synapse formation and maintenance, exemplify how retrograde neurotrophic signalling regulates network formation in the nervous system, a topic reviewed elsewhere 86, 87.

An additional role of neurotrophic signalling is the regulation of phenotypic specification of neurons. For example, in double *Bax* ^*−/−*^ */TrkA* ^*−/−*^ mice, peptidergic nociceptors that failed to innervate distal targets do not express markers of their lineage, such as calcitonin gene‐related peptide and substance P 74. Although the fate of this neuronal population is unclear, the absence of markers suggests a role of NGF on its phenotypic differentiation. NGF also regulates the phenotypic differentiation of nonpeptidergic nociceptors. In this cell population, Ret, the coreceptor for glial‐derived neurotrophic factor (GDNF), is expressed upon NGF stimulation. Ret induces the expression of the GDNF coreceptors GFRα1 and GFRα2, as well as other genes characteristic of the nonpeptidergic lineage. In the same neuronal subtype, NGF triggers the ERK1/2 pathway downstream to TrkA, and induces the expression of CBFβ, a cofactor of the Runx family of transcription factors, which upon binding to Runx1, promotes the nonpeptidergic nociceptor fate 88, 89. An example from the central nervous system is the phenotypic differentiation of basal forebrain cholinergic neurons. When their projections innervating the hippocampus are transected, thus inhibiting the target‐derived supply of NGF, these neurons stop the expression of cholinergic markers, such as choline acetyl transferase, as well as the neurotrophin receptors p75^NTR^ and TrkA. Although originally considered as evidence of cholinergic degeneration, the number of neurons remains unaltered and there is no evidence for neuronal apoptosis, suggesting that in this model, NGF is required for maintenance of phenotypic differentiation, but not for neuronal survival 90.

Strong evidence linking neurotrophin signalling to local protein synthesis and RNA transport is starting to emerge. For instance, *Impa1* mRNA is transported and translated in the axon in a NGF‐dependent manner, in sympathetic neurons grown in compartmentalised cultures. *Impa1* is one of the most abundant axonal transcripts, and its product (myoinositol monophosphatase‐1) is a key enzyme in the inositol pathway, which controls the cellular levels of phosphatidilinositol 91. NGF also promotes the transport of lamin B2 and Bcl‐w mRNAs (*Lmnb2* and *Bcl2 l2*) towards the axon. Both mRNAs are cotransported in RNA granules assembled by the RNA‐binding protein SFPQ. In this pathway, SFPQ acts downstream to NGF/TrkA, and its role is necessary for neurotrophin‐dependent axon viability 92. However, it is unclear whether these functions require the retrograde transport of signalling endosomes, or if they rely on local signalling. On the other hand, the NGF‐triggered delivery of β‐actin mRNA to the axon in cultured adult DRG neurons is dependent on retrograde signalling. This process requires the PI3K and MAPK pathways, and is impaired by the dynein inhibitor EHNA 93. Further studies are, however, needed to characterise the role of the retrograde neurotrophic signalling on mRNA transport and local translation, both in axons and dendrites.

### Roles in pathology

Neurotrophins and their receptors have been involved in the pathogenesis of a variety of diseases, including psoriasis 94, allergies 95, and cancer 96, 97. However, given their crucial role in neuronal homeostasis, deregulation or impairment of axonal transport of neurotrophin signalling endosomes is a common feature in many neurodegenerative conditions. Here, we present some examples highlighting the deficits in retrograde signalling in pathology, whilst a comprehensive coverage of this topic can be found elsewhere 20, 98.

Alzheimer's disease is a progressive neurodegenerative disorder characterised by memory loss and cognitive decline. The molecular hallmarks of this pathology include accumulation of extracellular plaques containing amyloid beta (Aβ) oligomers, and intracellular neurofibrillary tangles 99. Aβ oligomers reduce the transport of endosomes carrying BDNF‐GFP, through a mechanism replicated by the inhibition of the deubiquitinating enzyme UCH‐L1 100. In addition, increased expression of amyloid precursor protein or its proteolytic fragment C99 upregulates Rab5 activity, inducing enlargement of Rab5‐positive endosomes and reduced retrograde axonal transport of NGF in basal forebrain cholinergic neurons, which leads to cholinergic neuronal degeneration 101, 102. In this regard, clinical trials involving NGF gene delivery have been carried out, showing evidence of a trophic response (i.e. cholinergic axonal sprouting) that was detectable even 10 years after treatment 103.

Huntington's disease is caused by a polyglutamine (polyQ) expansion in huntingtin (Htt), and manifests with progressive cognitive, motor, and behavioural impairments that eventually become fatal 104. Although the multifaceted functions of wild‐type Htt are still matter of debate, it has been shown to form a complex with huntingtin‐associated protein 1 and the dynactin subunit p150^Glued^ 105. This complex is required for the retrograde transport of BDNF, a process inhibited by polyQ‐Htt, which leads to loss of neurotropic support and consequent neuronal toxicity 106. In agreement with this model, expression of polyQ‐Htt reduces the retrograde transport of TrkB‐BDNF complexes in dendrites of striatal neurons 107. In addition, reduction of wild‐type Htt expression inhibits the retrograde transport of Rab7‐positive vesicles in *Drosophila* larval axons 108.

Amyotrophic lateral sclerosis (ALS) is a progressive neurodegenerative disease affecting upper and lower motor neurons, which leads to muscle denervation and wasting, paralysis and death 109. The pathomechanisms at the basis of this disease are diverse; however, axonal transport defects are considered among the earliest disease phenotypes 21. One of the most characterised models of familial ALS is a mouse strain expressing a mutant form (G93A) of human superoxide dismutase 1 (SOD1) 110. Cultured primary motor neurons derived from SOD1^G93A^ mice present altered retrograde transport of signalling endosomes with fewer carriers, higher number of pauses, and oscillatory movements 111. This was confirmed *in vivo*, starting at presymptomatic stages of disease 19, 112. Interestingly, the defects in retrograde axonal transport in ALS precede the alterations reported in anterograde transport 113, 114 as well as other cellular pathological phenotypes, suggesting that alterations in retrograde transport may be a direct cause of this pathology, rather than a consequence. Interestingly, a form of familial early‐onset ALS is caused by mutations in the Rab5 GEF Alsin 115. Alsin loss of function deregulates the trafficking along the endolysosomal pathway in cultured motor neurons 116, and interferes with the transport of TrkB and insulin‐like growth factor 1 receptor in cortical and cerebellar granule neurons 117.

Charcot‐Marie‐Tooth disease (CMT) is the most common inherited peripheral neuropathy, affecting both sensory and motor nerves 118. CMT type 2B is caused by mutations in Rab7 that lead to a decreased nucleotide affinity and unregulated nucleotide exchange, without affecting its hydrolytic activity. These Rab7 mutants resemble the constitutively active Rab7^Q67L^; they exhibit enhanced interaction with a subset of Rab7 effectors, and are abnormally retained on target membranes 27, 119. CMT‐related Rab7 mutants affect the directionality of movement of Rab7‐positive endosomes, as well as the transport of carriers containing TrkA in NGF‐stimulated DRG cultures 120. The dynamics of Rab7‐positive endosomes is also altered in *Drosophila* and zebrafish CMT2B disease models, where a reduction of the pausing time has been reported for the Rab7 L129F, N161T, and V162M mutants 121, 122. The possibility that the impairment in axonal transport of NGF constitutes a more general pathological mechanism for other CMT subtypes has been recently explored in a study showing a reduction of velocity and an increase in pausing of retrograde NGF in a *Gars* ^*P234KY/+*^ model of CMT2D 123.

## A new organelle or a master of disguise?

### Formation and molecular signatures

**Figure 1:**
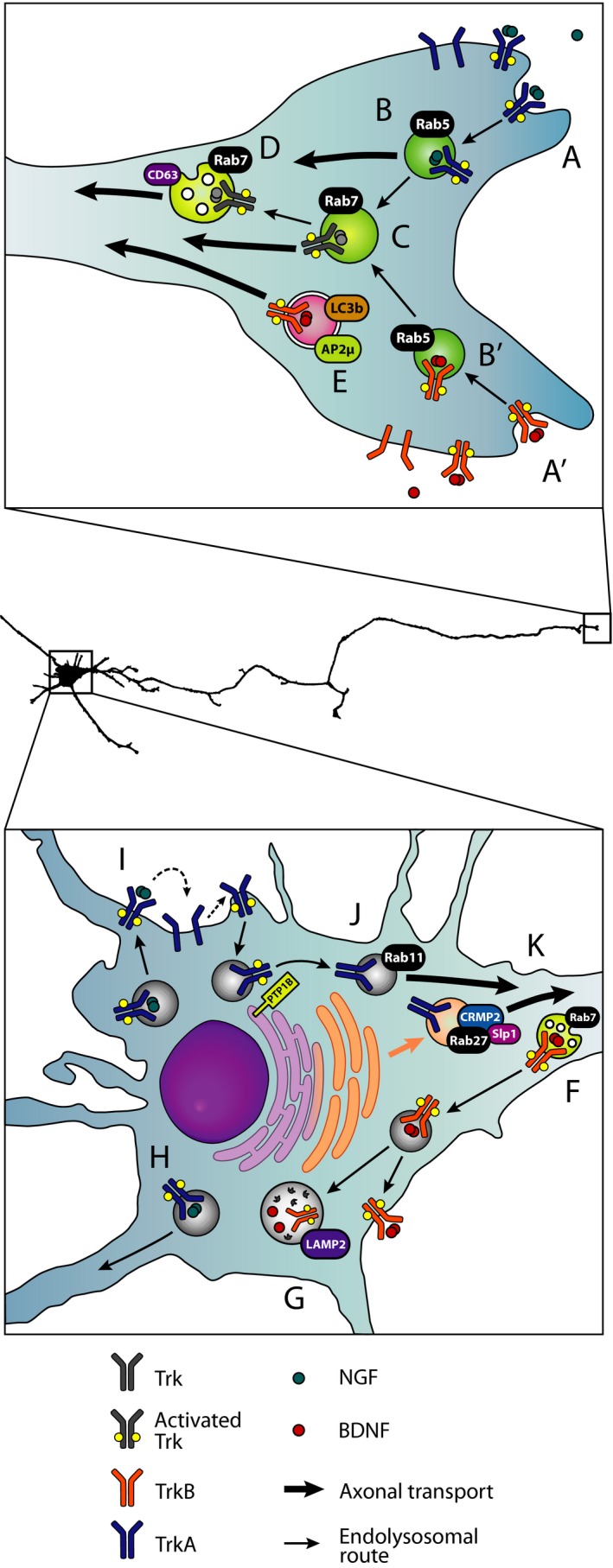
Retrograde neurotrophic signalling organelles and their main destinations after arrival to the cell body. Different types of signalling endosomes and their cellular destinations have been displayed on the top of a *camera lucida* trace of a mouse primary motor neuron in culture. The type of carriers and their sequential trafficking steps emerge from evidence collected using diverse experimental models, such as sensory, sympathetic, and motor neurons. Whether they are all present in every neuronal subtype is currently unknown. After being released by the target tissue, NGF and BDNF bind to TrkA (A) and TrkB (A’) respectively, triggering their phosphorylation and internalisation. TrkA‐NGF (B) and TrkB‐BDNF (B’) complexes enter the endocytic pathway and reach the early endosome, an organelle characterised by the presence of Rab5. Retrograde transport of Rab5‐positive carriers of TrkA‐NGF complex has been shown (thick arrow in B). Activated receptors are then sorted into a late endosome compartment (C), characterised by the presence of Rab7. This compartment can undergo retrograde axonal transport towards the soma, or progress along the endolysosomal route maturing into a multivesicular body (MBV; D), which contains both Rab7 and CD63. MVBs can also transport both receptor‐ligand complexes to the cell soma. In addition, autophagosomes have also been reported as retrograde carriers for TrkB‐BDNF (E). We have not included the origin of the autophagosome in the axonal tip, since it is still a matter of debate. After signalling endosomes reach the soma, there are sorted into a variety of destinations. Several lines of evidence indicate that neurotrophin receptors are delivered to somatic MVBs (F). TrkB‐BDNF in MVBs or single‐membrane vesicles can be either delivered to the plasma membrane or targeted to lysosomes for degradation (G). A subpopulation of TrkA‐NGF positive cargoes has been shown to be transported into dendrites (H), where it regulates synapse formation and maintenance. TrkA‐NGF complexes can also be recycled to the soma plasma membrane, where they might activate naïve TrkA receptors (I) and induce their recruitment to the anterograde axonal transport route in Rab11‐positive organelles (J). The activity of the endoplasmic reticulum‐resident phosphatase PTP1B is required to ensure that no activated TrkA is sorted into axons. In parallel, a pool of newly synthesised TrkA receptor is anterogradely transported in secretory vesicles characterised by the presence of Rab27, CRMP2 and Slp1 (K).

From a biogenesis perspective, the retrograde signalling carriers are unequivocally endosomes: they originate from the plasma membrane when neurotrophins bind their receptors and trigger their endocytosis (Fig. 1A,A’]). Internalisation of receptors is crucial not only for long distance propagation of neurotrophic effects, but also for engaging intracellular interactors enabling the activation of distinct signalling pathways 15, 124. It is critical for local effects relying on the accumulation of signalling molecules at specific locations, as in the case of synaptic assembly sites, branching points on neurites and the leading edge of a migrating precursor 51, 53, 84, 85, 125.

Specific mechanisms of internalisation have proved to be particularly important for generating intracellular compartments with distinct signalling capabilities. Using PC12 cells and DRG neurons, the group of William Mobley observed that upon NGF stimulation, recruitment of clathrin to the plasma membrane was increased, and TrkA receptors were rapidly endocytosed and sorted to small peripheral organelles together with NGF. Isolation of these endocytic vesicles showed that they were enriched in phosphorylated receptors together with clathrin heavy chain and its AP2 adaptor. Different signalling molecules, including PI3K, PLCγ and ERK1/2, were also present in the same samples 26. However, Halegoua and colleagues subsequently showed that clathrin‐dependent endocytosis accounted for only a fraction of internalised receptors in PC12 cells and DRGs, whilst a larger proportion was generated from macropinocytosis. They showed that sorting of TrkA to signalling carriers depends on the Rho‐GTPase Rac and the macropinocytic adaptor Pincher. These markers characterise a particular type of postendocytic organelle that, in contrast to signalling endosomes containing EGF receptors, was protected from lysosomal degradation 126, 127, 128. Interestingly, the mechanism of internalisation varies depending on the nature of the receptor, type of neuron, and ligand concentration. In this regard, Deinhardt and colleagues have shown that in primary motor neurons NGF stimulates the internalisation of p75^NTR^ and its targeting to the fast retrograde transport route by a mechanism initiated by the recruitment of p75^NTR^ to clathrin‐coated invaginations, which relies on dynamin, but not AP2 or AP180 adaptors 129.

After endocytosis, receptors are sorted to early endosomes characterised by the presence of the GTPase Rab5 (Fig. 1B,B’). Independent lines of evidence suggest that Rab5‐positive early endosomes are the most likely source of retrograde signalling endosomes. Mobley and coworkers have extensively characterised the ability of TrkA receptors to recruit specific signalling molecules to early endosomes, which regulate cellular responses, such as neurite outgrowth, in PC12 cells and DRG neurons 130, 131. They used isolated sciatic nerve to show that, after several hours of exposing the distal part of the nerve to NGF, signalling molecules including Rap1, PI3K, phosphorylated ERK1/2 and p38 were enriched in proximal sections of the axon, together with p75^NTR^, phosphorylated‐TrkA receptors and the early endosome markers Rab5 and EEA1 25. In addition, quantum dots‐labelled NGF internalised at DRG axon was found in organelles positive for TrkA, phosphorylated ERK1/2 and Rab5b 132.

The protein APPL1 is a Rab5 effector that has been shown to regulate the sorting of a subpopulation of early endosomes lacking EEA1 133, 134. The group of David Kaplan has shown that TrkA interacts with APPL1, suggesting that a specific pool of early endosomes can function as signalling endosomes. TrkA, APPL1, and GIPC1, a scaffolding protein that promotes trafficking of APPL1 vesicles to early endosomes, were found in endosomal fractions, particularly in axons of superior cervical ganglia neurons 135.

Notwithstanding the important role of early endosomes in the biogenesis and sorting of neurotrophin receptor‐containing carriers to the retrograde transport route, Deinhardt *et al*. found that, in spinal cord motor neurons, these organelles are characterised by the presence of Rab7 (Fig. 1C). By using H_C_T to label retrogradely transported endosomes, our laboratory has been able to describe a trafficking route shared by NGF, BDNF, p75^NTR^, and TrkB 41, 129. Upon clathrin‐dependent endocytosis, these cargoes are sorted sequentially first to a Rab5‐positive organelle that shows oscillatory movement in axons, and then to a Rab7‐positive compartment undergoing fast retrograde transport. In motor neurons, the initial sorting to an early endosome is a required step to reach fast retrograde axonal carriers, as was demonstrated by using dominant‐negative mutants of Rab5. Moreover, the axonal trafficking of Rab5 and Rab7 was shown to be similar in DRG neurons 41.

Importantly, the presence of Rab7 does not appear to target these organelles to degradation. During maturation from late endosomes to lysosomes, the vATPase drives the acidification of the endosomal lumen, providing the optimal environment for full cathepsin activity and promoting the dissociation of ligands from their receptors. The surprising finding that the vATPase is largely excluded from this compartment suggests that these organelles do not belong to the degradative pathway 136. Interestingly, similar Rab7‐positive retrograde organelles are involved in the transport of the P2X3 receptor in both motor and DRG neurons 137.

It is not clear whether Rab5‐positive axonal organelles have different degrees of mobility in other neuronal types. In hippocampal neurons, for example, it has been shown that a substantial amount of Rab5 is retrogradely transported, although its transport is not very progressive 138. However, when Zhou and colleagues followed fluorescent TrkB in the axon of cortical neurons, most of the receptor was in Rab7‐positive organelles and relied on Snapin to interact with DIC, thus engaging in retrograde axonal movement 36. Moreover, when H_C_T‐containing endosomes were purified from mouse embryonic stem cell‐derived motor neurons using paramagnetic iron beads at different time points after endocytosis, the amount of Rab5 associated to signalling endosomes progressively decreased, whilst Rab7 increased about 50% from 10 to 60 min upon internalisation, which is consistent with a sequential transit of the cargoes through Rab5‐ and Rab7‐positive compartments 24. Since the maturation of early endosomes to late endosomes depends on the switch between Rab5 and Rab7 49, these apparently controversial observations may be explained by the presence of a maturation gradient along the axon, which depends on the neuronal type and signalling context. In the work of Debaisieux and colleagues, other Rabs were also found associated to the purified retrograde carriers, suggesting that subpopulations of Rab7‐ and Rab5‐positive organelles bearing specific small Rab GTPases may constitute alternative pathways with distinct function(s) 24. Consistent with this hypothesis, it has been reported that the GTPase Rab11, generally associated to recycling cargoes directed to the plasma membrane, is also cotransported in TrkA‐containing retrograde carriers in sympathetic neurons 139, 140.

An alternative hypothesis about the nature of the retrograde signalling carriers is that they are multivesicular bodies (MVBs; reviewed in 141). These multiple‐membrane organelles (Fig. 1D) belong to the endolysosomal system 142 and have been shown to act as specialised transport organelles as well as a main source of exosomal vesicles 143. The possibility that MVBs may serve as retrograde carriers of activated neurotrophin receptors has been explored for almost four decades and it is based on the notion that specific cytoplasmic microenvironments are preserved in the lumen of the MVB. This would allow a snapshot of the signalling molecules present at the axon terminal at any particular time to be captured, transported, and made available in the soma 141. Recent evidence has strengthened the putative role of MVBs in targeting neurotrophin receptors to particular destinations. Consistent with the work showing that Pincher‐mediated internalised TrkB was sorted to MVBs in the soma of superior cervical ganglia neurons 126, the Bronfman group found that a pool of internalised p75^NTR^ is sorted to a compartment positive for CD63, a marker of MVBs, for its release in exosomes 144. Recently, Ginty's group reported the first evidence using live‐cell imaging that CD63 and TrkA are cotransported in retrograde carriers in the axon of sympathetic superior cervical ganglion neurons. Interestingly, the majority of axonal Rab5‐positive organelles exhibited short‐range bidirectional movement or were immobile, whereas Rab7‐organelles were most frequently retrograde. These organelles were positive for Lamp1, CD63, and RILP, and contained not only phosphorylated TrkA, but also other signalling molecules, including phosphorylated PLCγ. This group proposed that, after an initial sorting of the receptors to early endosomes in axon terminals, these transport organelles mature to MVBs that require Rab7 and its effectors, such as RILP, to engage retrograde transport. Some of the receptors would be exposed to the cytoplasm on the limiting membrane, helping to define the destination of these carriers, whereas the majority of receptors would be protected in the intraluminal vesicles. At their arrival in the cell body, MVBs would disassemble, originating single‐membrane vesicles with signalling potential 145. However, it is unlikely that RILP is the only downstream effector of Rab7 responsible for the coordination of retrograde transport, since a dominant‐negative mutant of RILP fails to alter the dynamics of H_C_T‐positive retrograde carriers in spinal cord motor neurons (G. Schiavo, unpublished results).

Other groups have suggested that a closely related organelle, the autophagosome, is the main retrograde carrier (Fig. 1E). Similarly to the MVBs, autophagosomes are multimembrane organelles involved in the degradation of a variety of organelles and cytoplasm 146. However, autophagosomes are generated by a distinct mechanism involving the elongation of a nucleating membrane to isolate a portion of cytoplasm or a specific organelle. A constitutive, nondegradative role has been suggested for autophagosomes by Erika Holzbaur and her group, who showed that in hippocampal neurons the generation of autophagosomes occurs most frequently at axon terminals, and it is independent of nutrient deprivation. These distally generated autophagosomes undergo retrograde transport and are not acidified until they reach the proximity of the cell body 147. A distinctive marker of this compartment is the protein LC3b, which participates in the elongation of the nascent phagophore. Recent work from the team of Volker Haucke using cortical and hippocampal neurons has shown that AP2μ interacts with LC3b and regulates the retrograde transport and maturation of autophagosomes *via* a mechanism independent of its established role as a clathrin adaptor. pTrkB is found in AP2μ‐ and LC3b‐positive organelles and a knockout mouse of AP2μ exhibited a decrease in both the retrograde transport of TrkB and dendritic complexity 148.

These partially conflicting observations point to an intrinsic diversity in the molecular nature of retrograde signalling carriers, whose relative abundance may be finely regulated depending on the neuronal type, developmental stage or signalling, and activity context.

### Complexity of membranes

An important morphological feature to help us to distinguish whether the retrograde signalling carriers are endosomes, autophagosomes or MVBs is their single‐ or multiple‐membrane nature. Early studies on the generation of signalling endosomes in PC12 cells showed that after Pincher‐mediated macroendocytosis, TrkA receptors were rapidly associated to MVBs 128. Interestingly, when subcellular fractionation was used to isolate NGF‐containing postendocytic organelles in PC12 cells, TrkA and p75^NTR^ were found in different populations of endosomal vesicles, translucent and electron dense, respectively. However, a small proportion of both receptors was also in MVBs 149. More recently, it has shown that 120 min after NGF‐induced internalisation, around 40% of p75^NTR^ is localised to MVBs, from which it can be released *via* an exosome‐mediated pathway 144.

Quantitative electron microscopy has played a key role to unravel the pleiotropic nature of retrograde signalling endosomes in neurons. The group of Campenot took advantage of a compartmentalised culture of sympathetic neurons to show that ^125^I‐NGF internalised in axons for 8–10 h was found in a variety of compartments, including smooth ER, small translucent vesicles, mitochondria, lysosomes, and MVBs 72, 150. The majority of the signal (37%) was associated to lysosomes, which accounted for 10% of the fractional area of the cell. Interestingly, MVBs were the most densely labelled organelles, concentrating a 6.7% of signal in just 0.4% of the area of the cell 150. Consistently, when the group of von Bartheld studied the retrograde transport of BDNF in motor hypoglossal neurons *in vivo*, they found that a large proportion of the BDNF was arriving to the soma and dendrites in MVBs 151. Crucially, when they examined axonal MVBs, they realised that organelles with multiple membranes were devoid of radiolabelled neurotrophic factors. In fact, most of the autoradiographic signal detected in axonal samples had no direct apposition nor spatial relationship with any observable organelles, suggesting that neurotrophins were associated to small vesicles that were masked by the autoradiographic precipitate. This observation was confirmed by using quantum dot‐conjugated BDNF 152.

Evidence showing that multiple‐membrane organelles are a common destination for retrogradely transported neurotrophic factors in the soma and dendrites appears to be consistent between cell lines and neuronal types, including superior cervical ganglia and hippocampal neurons 126, 127, 128 and embryonic stem cells‐derived motor neurons 153. Whether the signalling complexes are transported in multiple‐membrane organelles along the axon, however, is less clear from ultrastructural studies. Whereas the cited work of Altick and colleagues dismissed MVBs as retrograde carriers of neurotrophic factors 152, other authors have suggested a clear role for them. For example, the Meunier's team has used cholera toxin B subunit (CTB) in hippocampal neurons to label neurotrophic factor‐transporting organelles after axonal stimulation with BDNF in microfluidic devices, and found CTB‐positive multiple‐membrane organelle aligned with other single‐membrane vesicles in the axon 154. Working with a different *in vivo* model, the group of Hendry has proposed that MVBs are the main carriers of retrograde NGF and its receptors in sympathetic neurons 155.

Altogether, these findings suggest that neurotrophic signalling complexes are transported along the axon in organelles of different membrane complexity depending on the type of neuron, the nature of the neurotrophin and its concentration. An interesting possibility is that there is a gradual assembling of endocytic vesicles into multiple‐membrane organelles as they move from the axon ending towards the cell body. Supporting this hypothesis, a recent study from Ye and colleagues has shown using that TrkA receptors appear to be mainly in single‐membrane vesicles soon after internalisation in axonal tips of sympathetic neurons, whilst they are found predominantly in MVBs upon arrival to the proximal axon and cell body 145. Interestingly, the study of Altick in motor neurons also identified axonal MVBs as highly dynamic organelles and showed an example of vesicular fusion/fission in an axonal MVB 152.

Alternatively, based on the observation that MVBs contain a large proportion of retrogradely transported NGF accumulating in the soma 150, it can be hypothesised that MVBs are not transporting organelles, but are the end destination where signalling endosomes are targeted either to terminate the signal or to release the receptors in the extracellular medium *via* exosomes. Whether the formation of multiple‐membrane structures occurs in the axon of a particular subpopulation of neurons or it is restricted to the cell body, constitutes an important open question to be addressed to clarify the nature of these axonal retrograde carriers and their dynamics.

### Multiple destinations

What initially emerged as an answer for the problem of the propagation of neurotrophic signalling from the axon terminal to the soma is now becoming a more sophisticated matter: the spatiotemporal regulation of neurotrophic signalling in different domains of a living neuron at a particular stage of differentiation and under a certain physiological context.

The prevalent destination of the neurotrophic signal at any given time is probably the axon terminal. In sympathetic neurons, it is estimated that less than 10% of internalised TrkA is targeted to the retrograde transport route 156, 157, while the reminder pool recycles back to the terminal surface 158. Concentration of ligand and receptor at the axon terminal has been shown to be crucial to increase the sensitivity to neurotrophins and promote axon growth, either by local recycling of receptors or the recruitment of anterogradely transported TrkA 139. Whether or not this is also true for p75^NTR^, it is currently not clear. Nevertheless, it has been recently shown that after treatment of sympathetic neurons with NGF, TrkA receptors acting through the PI3K signalling pathway mediate the Arf6‐dependent fusion of intracellular vesicles containing p75^NTR^ to the plasma membrane, rapidly increasing its levels at the cell surface, and enhancing the sensitivity of neurons to NGF 159.

Retrogradely transported receptors that arrive to the soma have been proven to drive gene expression, survival, dendritic plasticity, and synaptic maintenance 36, 70, 84, 160. Many retrograde carriers containing activated receptors are going to persistently signal from late endosomes or other organelles until they fuse to lysosomes, as shown in Fig. 1F. Nevertheless, some of the functions in dendrites require the integrated control of local and global signalling, a matter that has been recently addressed by the Ginty's group by showing that in sympathetic neurons, a pool of retrogradely transported signalling endosomes were found in dendrites (Fig. 1G) and in close proximity to synapses 84. Importantly, the majority of these organelles (~ 80%) remained mobile, whilst the soma‐bound endosomes are less dynamic, suggesting that these organelles are different 84. Recent work from the group of Bettina Winckler explored the heterogeneity of retrograde signalling endosomes after their arrival to the cell body. These authors showed that, both in cultured neurons and *in vivo*, TrkA receptors labelled at the distal axon are found in somatic organelles that were positive for a variety of endosomal markers, including Rab7, Rab11, and EEA1. At molecular level, they found that a large proportion of the retrogradely transported TrkA in dendrites was targeted to the cell surface either in the soma or dendrites, prior to being sorted to different populations of endosomes 140. This novel sorting mechanism may regulate the different outputs of activated receptors, leading to dendrite‐to‐nucleus signalling 161 or local actions (e.g. dendrite branching 125).

Finally, it is also possible that receptors that have been transported from the axonal tip to the soma are targeted to anterograde transport 162. It had been previously shown that neurotrophic signalling originated from distal axons can promote anterograde Trans‐cytosis of TrkA and TrkB receptors from the cell body, increasing sensitivity to ligand at the axon terminal 139, 163. Recent work from the group of Rejji Kuruvilla showed that in sympathetic neurons, a population of retrogradely transported TrkA is inserted on the plasma membrane at the soma (Fig. 1H), where they can promote phosphorylation and endocytosis of resident receptors. Once internalised, TrkA receptors are dephosphorylated by the PTP1B phosphatase before being transported anterogradely to the axon terminal (Fig. 1I), suggesting a mechanism that allows retention of the phosphorylated pool of receptors in the somato‐dendritic compartment, where they can engage persistent signalling, whereas only inactive receptors are sent back to the axon to regulate distal neurotrophin sensitivity. Importantly, conditional deletion of PTP1B in *NGF* ^*+/−*^ mice reduced axonal TrkA levels, neuron survival and target innervation, emphasising the relevance of this mechanism *in vivo*, especially under limited availability of NGF 162.

Given the diversity of destinations, and the cellular and functional outputs of retrograde signalling carriers, an important unaddressed question is whether the molecular signatures acquired during the biogenesis of these organelles determine their final destination, or whether the diversification of these carriers occurs at their arrival in the cell body as a function of distinct signalling contexts.

## Concluding remarks: diversity matters

It is clear from the evidence herein presented that a variety of axonal retrograde signalling carriers, which differ in their molecular composition, are found in neurons. They are also diverse in terms of biogenesis and membrane complexity – some of them have been characterised as conventional endosomes, whilst others have been described as MVBs or autophagosomes.

These diverse observations can be explained at least in part by differences in experimental models, for example, the combination of receptors expressed by different neuronal types, and the specific neurotrophic factor under investigation. Results may be obtained from the same cellular type but at different stages of neuronal maturation, or focussing on different areas of axon and dendrites. In addition, multiple approaches have been used to label and monitor the dynamics of signalling endosomes: radioactive and/or fluorescent neurotrophins, antibodies binding the receptors, toxin fragments and viruses that share the same transport route of the neurotrophic factors, to mention a few. Although these different scenarios are likely to contribute to the different results accumulating in the literature, the studies discussed in this review reveal an intrinsic heterogeneity in the organelles carrying neurotrophic signals along the axon. Whether these organelles are separated compartments with specific cellular destinations and functions, or whether they are just different maturation stages of a highly dynamic and flexible network of intracellular membrane‐bound vesicles remain to be addressed.

Altogether, we believe that the available data tend to disregard the idea of signalling endosomes as an entirely new class of organelles with distinctive molecular signatures. Rather, they indicate that trafficking of neurotrophin receptors exploits multiple routes to ensure not only efficient and flexible communication between distal portions of axons and the cell body, but also a precise regulation of the spatiotemporal dynamics and compartmentalisation of signalling cascades. In this regard, one of the biggest challenges is to explain the diversity of destinations of the retrograde signalling endosomes after arrival to the soma. The implementation of new tools for live imaging of axonal transport in intact tissue 164, as well as the possibility of purifying and performing quantitative proteomics of subpopulations of retrograde carriers, shall be critical to reveal the differences in cargoes, adaptors, and signalling molecules that characterise different functional subpools, and to resolve the mechanisms of their assembly and disposal.
